# The association of a reduced susceptibility to moxifloxacin in causative *Clostridium* (*Clostridioides*) *difficile* strain with the clinical outcome of patients

**DOI:** 10.1186/s13756-020-00765-y

**Published:** 2020-06-30

**Authors:** Marcela Krutova, Vaclav Capek, Elka Nycova, Sabina Vojackova, Magda Balejova, Lenka Geigerova, Renata Tejkalova, Lenka Havlinova, Iva Vagnerova, Pavel Cermak, Lenka Ryskova, Petr Jezek, Dana Zamazalova, Denisa Vesela, Alice Kucharova, Dana Nemcova, Martina Curdova, Otakar Nyc, Pavel Drevinek

**Affiliations:** 1 Department of Medical Microbiology, Charles University, 2nd Faculty of Medicine and Motol University Hospital, Prague, Czech Republic; 2 Bioinformatics centre, 2nd Faculty of Medicine, Charles University, Prague, Czech Republic; 3 Department of Medical Microbiology, Hospital Bulovka, Prague, Czech Republic; 4 Department of Medical Microbiology, University Hospital Brno, Brno, Czech Republic; 5 Department of Medical Microbiology, Hospital Ceske Budejovice, Ceske Budejovice, Czech Republic; 6 Department of Medical Microbiology, Faculty of Medicine, Charles University and University Hospital Plzen, Plzen, Czech Republic; 7 Department of Medical Microbiology, Faculty of Medicine, Masaryk University and St. Anne’s University Hospital, Brno, Czech Republic; 8 Department of Medical Microbiology and Immunology, Hospital Liberec, Liberec, Czech Republic; 9 Department of Microbiology, Faculty of Medicine and Dentistry, Palacky University Hospital, Olomouc, Czech Republic; 10 Department of Medical Microbiology, Thomayer’s Hospital, Prague, Czech Republic; 11 Department of Clinical Microbiology, University Hospital Hradec Kralove, Hradec Kralove, Czech Republic; 12 Department of Clinical Microbiology and Parasitology, Hospital Pribram, Pribram, Czech Republic; 13 Department of Clinical Microbiology, Hospital Nove Mesto na Morave, Nove Mesto na Morave, Czech Republic; 14 Department of Medical Microbiology, Hospital Jindrichuv Hradec, Jindrichuv Hradec, Czech Republic; 15 Department of Medical Microbiology, Hospital Tabor, Tabor, Czech Republic; 16 Department of Clinical Microbiology, Institute for Clinical and Experimental Medicine, Prague, Czech Republic; 17 Department of Clinical Microbiology, Military University Hospital, Prague, Czech Republic

**Keywords:** *Clostridium difficile* infection, *Clostridioides difficile* infection, Czech Republic, PCR ribotype 001, PCR ribotype 176, Moxifloxacin, Mortality

## Abstract

**Objectives:**

To investigate the relationship between *Clostridium (Clostridioides) difficile* strain characteristics and *C. difficile* infection (CDI) outcome.

**Methods:**

Between October and December 2017, 16 hospitals collected epidemiological data according to the European Centre for Disease Prevention and Control (ECDC) surveillance protocol for CDI. *C. difficile* isolates were characterized by ribotyping, toxin genes detection and antibiotic susceptibility testing to metronidazole, vancomycin and moxifloxacin.

**Results:**

The overall mean CDI incidence density was 4.5 [95% CI 3.6–5.3] cases per 10,000 patient-days. From the 433 CDI cases, 330 (76.2%) were healthcare-associated, 52 (12.0%) cases were community-associated or of unknown origin and 51 (11.8%) CDI cases recurrent; a complicated course of CDI was reported in 65 cases (15.0%). Eighty-eight (20.3%) of patients died and 59 of them within 30 days after the CDI diagnosis.

From the 379 *C. difficile* isolates, the most prevalent PCR ribotypes were 001 (*n* = 127, 33.5%) and 176 (*n* = 44, 11.6%). A total of 186 (49.1%) isolates showed a reduced susceptibility to moxifloxacin (> 4 mg/L) and 96.4% of them had Thr82Ile in the GyrA. Nineteen isolates revealed reduced susceptibility to metronidazole and two isolates to vancomycin (> 2 mg/L).

A fatal outcome was associated with a reduced susceptibility to moxifloxacin, the advanced age of the patients and a complicated course of CDI (*p*<0.05). No association between ribotype, binary toxin and a reduced susceptibility to moxifloxacin and complicated course or recurrent CDI was found.

**Conclusions:**

A reduced susceptibility to moxifloxacin, in causative *C. difficile* strains was associated with fatal outcome of the patients, therefore it is an important marker in surveillance of CDI.

## Introduction

*Clostridium difficile*, recently reclassified as *Clostridioides difficile* [1], is a leading pathogen of gastrointestinal infections in hospitalised patients [2]. Between 2005 and 2013, several European multicentre studies aimed at mapping *C. difficile* epidemiology in Europe and an increase of CDI incidence density was found to be concomitant with changes in prevailing ribotypes; however, the design of the studies varied [3].

To standardize CDI epidemiology data collection, the European centre for disease prevention and control (ECDC) released a surveillance protocol with three options of CDI surveillance intensity (“minimal, light and enhanced”). The “minimal option” collects hospital-level CDI data, the “light version” collects also CDI case-based data, including data on mortality. The “enhanced” option collects hospital data, CDI case based data and microbiological data. Microbiological data includes results on *C. difficile* isolates ribotyping, the presence of toxins A/B or toxin genes and antimicrobial susceptibility testing to metronidazole, vancomycin and moxifloxacin [3]. While metronidazole and vancomycin are recommended as CDI treatment drugs [4], a reduced susceptibility to moxifloxacin a fluoroquinolone class of drug, is suggested as an epidemiological marker for certain *C. difficile* ribotypes spread in healthcare settings [5].

After a successful pilot testing of all three CDI surveillance levels in 2013 [6], 20 countries participated in the first European standardized CDI surveillance wave in 2016 [7]. The “enhanced” version of the CDI surveillance protocol was used in 44.0% (*n* = 261) of all hospital surveillance periods (*n* = 593) but almost half of these data (118 periods) came from one country, Belgium. Moreover, characteristics of *C. difficile* isolates were not available for all enhanced case based data [7].

Therefore, we aimed to collect complete CDI surveillance data according to the “enhanced level” of ECDC surveillance protocol for CDI and to investigate the relationship between *C. difficile* strain characteristics and CDI outcome.

## Material and methods

### Study protocol

Between October and December 2017, 16 hospitals submitted epidemiological data on CDI patients, according to the ECDC surveillance protocol v 2.3 [8], and sent *C. difficile* isolates for further characterisation. Eight hospitals are tertiary care institutions, seven secondary care facilities and one hospital is a specialised organ transplant centre. The surveillance period covered a total of 16,109 hospital-beds, which was 20.1% of the hospital-bed capacity in the Czech Republic in 2017 [9].

The unformed stool sample of patients suspected of having CDI were tested at microbiological departments of participating hospitals based on a physician’s request. All but one of the participating hospitals used a sensitive screening test, glutamate dehydrogenase, followed by the detection of toxins A/B. Of those, ten hospitals used the nucleic acid amplification test (NAAT) or a toxigenic culture for further investigation of GDH positive and toxins A/B negative stool samples, while five hospitals, performed a *C. difficile* culture of GDH positive and toxin A/B negative samples but did not confirm the presence of toxin genes or toxins A/B in those *C. difficile* cultures. One hospital used a nucleic acid amplification test as a stand-alone test, which is not currently recommended [10].

### *Clostridium difficile* culture and isolates characterisation

In *C. difficile* isolates, a capillary-electrophoresis ribotyping was performed according to the consensus protocol [11]. The presence of toxin genes (*tcdA*-toxin A, *tcdB*-toxin B, *ctdA* and *cdtB* – binary toxin) was investigated by multiplex PCR [12]. The antibiotic susceptibility of the isolates to metronidazole, vancomycin and moxifloxacin was determined by the agar dilution method on Wilkins-Chalgren agar (Oxoid). The epidemiological cut-off values (ECOFFs) for metronidazole (2 mg/L), vancomycin (2 mg/L) and moxifloxacin (4 mg/L) were applied according to the European Committee on Antimicrobial Susceptibility Testing (EUCAST). The quinolone resistance-determining region (QRDR) in the *gyrA* gene was amplified and sequenced to investigate the molecular mechanism of reduced susceptibility to moxifloxacin [13], Supplementary data.

### Statistical analysis

Differences between groups were evaluated using χ2 test or Fisher exact test for categorical variables and the Wilcoxon test for continuous variables. A univariate logistic regression model was used for the analyses of associations between individual variables and outcomes of interest. P-values were adjusted for multiple comparisons using a Holm-Bonferroni method. A *p*-value of ≤0.05 was considered statistically significant. Analyses were conducted using the R statistical software version 3.5.1 [14]..

The mean CDI incidence density was calculated from the CDI incidence densities of each participating hospital. The patient’s outcome was followed until the patient was discharged from hospital or died. There was no post-discharge follow up regarding the readmission or death of patients.

## Results

### *Clostridium Clostridioides difficile* infections

During the three-month surveillance period, 433 CDI cases were recorded in 16 hospitals. The average age of patients was 69.1 years (median 74 years) and 52% were females. The mean testing frequency was 54.0 [95% CI 44.8–63.2] tests per 10,000 patient days. The mean CDI incidence density was 4.5 [95% CI 3.6–5.3] cases per 10,000 patient-days. Of 433 reported CDI cases, 330 (76.2%) were healthcare associated, the incidence density 3.5 [95% CI 2.8–4.1] cases per 10,000 patients-days. The origin of CDI at the same hospital was reported in 319 cases and at another hospital in 11 cases. Thirty-six cases (8.3%) were community-associated and in 16 cases (3.7%) the origin of CDI was unknown; the common-incidence density was 0.5 [95% CI 0.3–0.8] cases per 10,000 patients-days. Fifty-one CDIs (11.8%) were recurrent, with an incidence density of 0.5 [95% CI 0.3–0.7] cases per 10,000 patients-days.

A complicated course of CDI (admission for CDI from the community; admission to an intensive care unit; surgery for toxic megacolon or death) was reported in 65 cases (15.0%). Eighty-eight patients (20.3%) died and of those, 28 patients had a complicated course of CDI (*p* = 0.00). Fifty-nine patients died within 30 days after CDI diagnosis and of those, 21 patients had a complicated course of CDI (*p* = 0.00).

Data on CDI by origin, course and outcome are summarised in Table 1.

**Table 1:** Overall results from 3-months CDI surveillance in the Czech Republic; *CDI Clostridium difficile* infection, *CI* confidential interval

*Clostridium (Clostridioides) difficile* infections (CDI) by type, 16 hospitals, 10–12/2017	Number (%)	Mean incidence density (95% CI)
CDI cases	433 (100)	4.5 [95% CI 3.6–5.2]
Healthcare-associated CDIs	330 (76.2)	3.5 [95% CI 2.8–4.1]
Community-associated and unknown origin CDIs	52 (12.0)	0.5 [95% CI 0.3–0.8]
Recurrent CDI	51 (11.8)	0.5 [95% CI 0.3–0.7]
Complicated course	65 (15.0)	
Death	88 (20.3)	
Characterisation of *C. difficile* isolates, 16 hospitals, 10–12/2017	379 (87.5)	
Ribotyping and toxin genes detection		
Ribotype 001	127 (33.5)	
Ribotype 176	44 (11.6)	
Others (30 profiles)	208 (54.9)	
Binary toxin genes positive	75 (19.8)	
Antimicrobial susceptibility testing	Susceptible (%)	Reduced susceptibility (%)
Metronidazole (breakpoint 2 mg/L)	360 (95.0)	*n* = 19 (5.0), RTs: 001 *n* = 8, 027 *n* = 6, 176 *n* = 5
Vancomycin (breakpoint 2 mg/L)	377 (99.5)	*n* = 2 (0.5), RTs: 001, 012 *n* = 1, each
Moxifloxacin (breakpoint 4 mg/L)	193 (50.9)	*n* = 186 (49.1), RTs: 001 *n* = 114, 002 *n* = 1, 012 *n* = 8, 017 *n* = 1, 027 *n* = 9, 033 *n* = 1, 078 *n* = 2, 126 *n* = 3, 176 *n* = 44, unrecognized *n* = 3

### Microbiological data

Of the 433 CDI cases, 379 *C. difficile* isolates (87.5% of CDI cases) were available for further characterisation. The most frequently found ribotypes were 001 (*n* = 127, 33.5%, detected in 16/16 hospitals) and 176 (*n* = 44, 11.6%, 11/16 hospitals). The remaining 208 isolates (54.9%) belonged to 30 different ribotyping profiles. The prevalence of 5% was exceeded only by RTs: 014 (*n* = 24; 6.3%) and 012 (*n* = 23; 6.1%).

All the 379 *C. difficile* isolates carried the *tcdA* gene (toxin A), and 378 isolates also carried the *tcdB* (toxin B); one *C. difficile* isolate of the ribotype 033 was *tcdB* gene negative. Seventy-five (19.8%) of the *C. difficile* isolates (RTs: 023, 027, 033, 078, 126, 176 and two unrecognized profiles) also harboured the binary toxin genes (*cdtA* and *ctdB*).

A reduced susceptibility to metronidazole was observed in 19 isolates (RTs: 001 *n* = 8, 027 *n* = 6, 176 *n* = 5, the minimum inhibitory concentrations (MICs) were (3–8 mg/L) and an elevated MIC, reaching the breakpoint of 2 mg/L, was observed in 47 *C. difficile* isolates (RT176 *n* = 16; RT001 *n* = 25; RT027 *n* = 2; others *n* = 4). An elevated MIC and reduced susceptibility to metronidazole was associated with RTs 001 and 176 (*p* = 0.00, 0.00; *p* adjusted = 0.03 and 0.00, respectively). A reduced susceptibility to vancomycin was found in two isolates (RTs 001 and 012; MIC 3 mg/L).

Although a reduced susceptibility of the *C. difficile* isolates to metronidazole and vancomycin was rarely found, a reduced susceptibility to moxifloxacin was observed in 186 *C. difficile* isolates (49.1%; RTs: 001, 002, 012, 017, 027, 033, 078, 126, 176, one unrecognized) and from those, 179 *C. difficile* isolates (96.2%) showed Thr82Ile in the GyrA. Thirty *C. difficile* isolates susceptible to moxifloxacin used as controls were wild types.

Data on the characterisation of *C. difficile* isolates are summarized in Table 1.

### Clinical data analysis

In our study, the older patients were more likely to be infected with the *C. difficile* ribotype 001 (Mean 72.7 vs 66.8 years; Median 76.0 vs 72.0 years; *p* = 0.00, p adjusted = 0.02) and/or with *C. difficile* strain showing a reduced susceptibility to moxifloxacin (*p* = 0.00, p adjusted = 0.00).

All-cause mortality was associated with advanced age (OR = 1.0; 95% CI 1.0–1.0; *p* = 0.00), a complicated course of CDI (OR = 4.2; 95% CI 2.4–7.4; *p* = 0.00), RT001 infection (OR = 0.4; 95% CI 0.3–0.7; *p* = 0.00), and/or *C. difficile* strains with a reduced susceptibility to moxifloxacin (OR = 2.9; 95% CI 1.7–4.9; *p* = 0.00).

The 30-days mortality was associated with advanced age (OR = 1.0; 95% CI 1.0–1.0; p = 0.01), a complicated course of CDI (OR = 4.5; 95% CI 2.4–8.4; *p* = 0.00), and/or reduced susceptibility to moxifloxacin in CDI causative strains (OR = 2.4; 95% CI 1.3–4.3; *p* = 0.00).

No association between age, gender, CDI origin, RT 001 or 176, binary toxin genes, a reduced moxifloxacin susceptibility, recurrent CDIs and a complicated course of CDI was found in this study.

Statistical analyses are summarised in Tables 2 and 3.

**Table 2:** Univariate comparison of the baseline characteristics of *C. difficile* infections caused by *C. difficile* strains of ribotype 001, ribotype 176, binary toxin genes carrying and showing the reduced susceptibility to moxifloxacin

	Ribotype 001		Ribotype 176		Binary toxin genes		Moxifloxacin (RS)	
	*p*-value	Adjusted *p*-value	*p*-value	Adjusted *p*-value	*p*-value	Adjusted *p*-value	*p*-value	Adjusted *p*-value
Age	0.00	0.02	0.30	1.00	0.24	1.00	0.00	0.00
Gender	0.16	0.95	0.02	0.18	0.03	0.23	0.39	1.00
HA CDI origin	0.03	0.23	1.00	1.00	0.96	1.00	0.02	0.22
Recurrent CDI	0.35	1.00	0.09	0.61	0.26	1.00	0.35	1.00
Complicated CDI	0.27	1.00	0.12	0.86	0.90	1.00	0.93	1.00
30-days mortality	0.10	0.40	0.40	1.00	0.94	1.00	0.01	0.04
All-cause mortality	0.00	0.01	0.67	1.00	0.87	1.00	0.00	0.00

*CDI Clostridium difficile* infection, *CI* confidential interval, *HA* healthcare-associated, *RS* reduced susceptibility

**Table 3:** Unadjusted analysis of selected predictors for 30-days mortality and all-cause mortality

	30 - days mortality			All- cause mortality		
	OR	95% CI	*p*-value	OR	95% CI	*p*-value
Ribotype 001	0.6	0.3–1.1	0.07	0.4	0.3–0.7	0.00
Ribotype 176	0.7	0.3–1.4	0.29	0.8	0.4–1.6	0.53
Binary toxin genes	1.1	0.5–2.2	0.80	0.9	0.5–1.7	0.75
Moxifloxacin (RS)	2.4	1.3–4.3	0.00	2.9	1.7–4.9	0.00
Age	1.0	1.0–1.0	0.01	1.0	1.0–1.0	0.00
Gender	2.0	1.1–3.5	0.02	1.5	0.9–2.4	0.11
CDI Origin	1.8	0.5–6.2	0.33	1.6	0.6–4.4	0.32
Complicated CDI	4.5	2.4–8.4	0.00	4.2	2.4–7.4	0.00

*CDI Clostridium difficile* infection*, CI* confidential interval*, OR* Odds ratio*, RS* reduced susceptibility

## Discussion

In the Czech Republic, the CDI incidence density was shown to have a downward trend when compared to studies performed in 2014 (6.1 CDI cases per 10,000 patient-days) [15] and 2015 (5.2 CDI cases per 10,000 days) [16]. A further reduction to 3.9 cases per 10.000 patient-days was found in 2016 when the ECDC standardized protocol was adopted in 19 Czech hospitals [7]. In the currently reported surveillance period (10–12/2017), similar mean CDI incidence density rates were observed as in the three-month period of 2016 (4.5 vs 3.9 per 10,000 patients-days) [7]. However, it is of note that although the number of hospitals participating in the current study was lower than in 2016 (16 vs 19), the number of patient-days was higher (1,029,834 vs 924,021), respectively [7].

The proportion of RTs 001 and 176, compared to other detected RTs, showed an increase of RT001 between 2014 and 2015 (24 and 33.5%) [15, 16] and a decline in RT176 for 2014, 2015 and 2017 (29.1%; 25.5 and 11.6%) [15, 16]. The proportion of RT001 in this study is the same as in 2015 (33.5%) [16].

Importantly, the *C. difficile* isolates belonging to RTs 001 and 176 from this study were associated with elevated MICs and/or a reduced susceptibility to metronidazole (MIC≥2 mg/L). Currently, in Europe, metronidazole is recommended as a first-line CDI treatment drug for initial and non-complicated CDI [4] but, due to the growing evidence of its limited efficacy, vancomycin was suggested as a replacement for metronidazole in the CDI treatment algorithm [17]. This is in agreement with the Infectious Diseases Society of America (IDSA) and the Society for Healthcare Epidemiology of America (SHEA) guidelines [18]. In addition, two *C. difficile* isolates in this study showed a reduced susceptibility to vancomycin (MIC = 3 mg/L) yet when the patients were treated with vancomycin they improved clinically and both were discharged from hospital.

A reduced susceptibility to moxifloxacin was observed in 49.1% of the *C. difficile* isolates. Prior to this study, a high proportion of isolates that showed a reduced susceptibility to moxifloxacin was reported in European *C. difficile* isolates collected during the ClosER study, 7/2011–7/2014 (35.8%) [19] and in the standardized CDI surveillance in 2016 (363/523; 69.4% cases with data on susceptibility) [7].

The spread of fluoroquinolone-resistant *C. difficile* strains is thought to be associated with selective pressure caused by the frequent prescription of fluroquinolones [5]. However, this is unlikely to be the only contributory factor. As observed by Vernon et al., fluoroquinolone resistance in *C. difficile* can affect bacterial fitness, in both benefit and burden, depending on the causative mutation. Thr82Ile, the most prevalent mutation in our study, exhibited a significant fitness advantage in competitive batch culture and a higher mutant-to-parent ratio in a co-culture chemostat model [20]. The fluoroquinolone resistance caused by Thr82Ile plays undoubtedly a significant role in the global spread of RT027 [21]. The impact of the Thr82Ile-mediated reduced susceptibility to fluroquinolones on the epidemiology of CDI is also supported by the detection of Thr82Ile in European *C. difficile* isolates with a reduced susceptibility to moxifloxacin that showed clustering within–country and are tightly-clustered geographically [22].

The hospital mortality measured in our study was at a similar level to the latest European data (20.3% vs 20.7%), [7]. Several studies aimed to identify the strain specific characteristics, i.e. ribotype or the presence of binary toxin genes, linked adversely to patient outcomes [23–25] and the most common ribotype found to be associated with mortality was ribotype 027 [23, 24]. Although RT176 is genetically related to the ‘hypervirulent’ RT027 [26], our data did not support the relationship between RT176 CDI and a poorer outcome for the patients. These data are in agreement with data on the outcome of patients infected by RTs 027 and 176 collected during the pilot European standardized CDI surveillance in 2013 [6]. Whereas in our study, RT001 CDIs were shown to be associated with all-cause mortality, this statistically significant association was not confirmed when mortality within 30-days was analysed. In addition, no association between 30-days mortality and RT001 CDI was found in the UK study, where RT001 represented 16.8% of CDI cases and 25.4% of deaths [24].

Our findings did not support the relationship between mortality and a particular ribotype, nor did we confirm the recent observation on the effect of the presence of binary toxin genes on all-cause mortality [25]. In the study of Berry et al. [25], more than a half of 213 binary toxin positive *C. difficile* isolates were RTs 027 and 078 (*n* = 99 and 65, respectively) frequently referred to as ‘hypervirulent’ [23, 24, 27], whereas in our study the majority of binary toxin gene positive isolates (*n* = 75) belonged to RT176; the RTs 027 and 078 were represented by 9 and 12 isolates respectively. Similar to the study of Reigadas et al. [28], our results showed no association between the presence of binary toxin genes and recurrence or mortality in non-027 *C. difficile* isolates.

From the *C. difficile* strain characteristics studied here, a reduced susceptibility to moxifloxacin in *C. difficile* isolates was identified as a risk factor for all-cause mortality and 30-days mortality, therefore we believe it can be considered as a factor of pathogenicity per se. Although a reduced susceptibility to moxifloxacin is widely distributed in *C. difficile* clinical strains, the effect of this phenotype on patients’ outcome has not been reported previously. In contrary to Gram-negative bacteria, fluoroquinolone resistance was found to be a risk factor for in-hospital mortality, bacteremia or sepsis caused by fluoroquinolone resistant *E. coli* [29, 30].

The present study has several limitations. It is a national multicentre study and thus the results can be affected by the endemic occurrence of the two ribotypes 001 and 176 in the Czech Republic. Further, data on fatal cases could have been incomplete as, in the surveillance protocol used in this study, there was no post-discharge follow-up. Further data are needed in order to evaluate the role of a reduced susceptibility to moxifloxacin in *C. difficile* strains on CDI epidemiology and the outcome of patients with CDI.

## Conclusion

In our study, the amino acid substitution Thr82Ile in the GyrA was the most common molecular mechanism for a reduced susceptibility to moxifloxacin in *C. difficile* isolates. A reduced susceptibility to moxifloxacin, in causative *C. difficile* strains was associated with fatal outcome of the patients, therefore it is an important marker in surveillance of CDI.

## Supplementary information

**Additional file 1.**

**Additional file 2.**
